# Optimization of a polymerase chain reaction based assay for the detection of malaria in human saliva samples

**DOI:** 10.1186/1475-2875-9-S2-P38

**Published:** 2010-10-20

**Authors:** Ofentse J Pooe, Addmore Shonhai, Sungano Mharakurwa

**Affiliations:** 1 Department of Biochemistry and Microbiology, University of Zululand, Private Bag 1001, Kwa Dlangezwa, 3886, South Africa; 2 The Malaria Institute at Macha, P.O. Box 630166, Choma, Zambia; 3 Department of Molecular MIcrobiology & Immunology, Johns Hopkins Bloomberg School of Public Health, 615 N. Wolfe Street, Baltimore MD21205, USA

## Background

Malaria is responsible for more than 1 million deaths per year; most of the victims are young African children [1]. Recent studies have found that *Plasmodium falciparum* DNA is detectable in human saliva as in human blood [2]. However the nature of the source of the saliva fraction that serve as source of this DNA still unclear. Thus this study sought to establish the constituents of human saliva that harbours parasite the DNA in malaria infected subjects.

## Method

To determine whether the source of malaria amplifiable DNA in saliva was intracellular or free DNA in saliva, different saliva extractions were used to extract DNA; i.e. the pellet only , supernatant only and pellet and supernatant mixture. DNA was extracted using the Qiagen DNEasy^®^ kit on different saliva fractions from 46 malaria positive (thick film positive & blood PCR positives) individuals and 42 malaria negative individuals were used in this study. Saliva DNA extracts were subjected to nested PCR in separate batches according to their DNA fractions.

## Results

PCR conducted on both blood and saliva (pellet fraction) was more sensitive than conventional microscopy conducted on thick blood films. The pellet fraction was a more reliable source of amplifiable parasite DNA compared to the soluble saliva fraction. However it was noted that by reconstituting the pellet fraction with an aliquot of the supernatant, detection sensitivity from the pellet fraction could be improved. Through optimizing the PCR recipe we were able to increase the PCR amplification using saliva DNA extracts to 95, 65% sensitivity, with PCR on blood as the gold standard.

## Conclusion

This study further confirms that saliva is a reliable source for detecting malaria based methods and could potential be used as an alternative non-blood based and non invasive malaria detection assay. Furthermore this study established that DNA is more enriched in the pellet fraction compared to the insoluble fraction of saliva. Future work could involve optimization of this assay to make it cheaper and more robust for use in malaria surveillance.
